# An empirical study on the influence of new generation employees’ job satisfaction on contextual performance in the energy industry

**DOI:** 10.1016/j.heliyon.2024.e30073

**Published:** 2024-04-24

**Authors:** Hu Xiao, Wei Xie, Bo Liu

**Affiliations:** a Department of Admissions and Employment, Southwest Petroleum University, Chengdu, 610500, China; b School of Economics and Management, Southwest Petroleum University, Chengdu, 610500, China

**Keywords:** New generation employees in the energy industry, Job satisfaction, Organizational commitment, Self-efficacy, Contextual performance

## Abstract

New generation employees in the energy industry generally suffer from several problems such as poor psychological endurance, lack of professional literacy, excessive mental stress, and low labor enthusiasm, which will affect the employee's individual contextual performance and stability of the labor market. In the context of energy transition, therefore, it is of practical significance to solve their problems in routine work and identify the factors affecting the contextual performance of next generation employees in the energy industry. In this paper, the theoretical model with job satisfaction as an independent variable, organizational commitment as a mediator, the sense of self-efficacy as a moderator, and contextual performance as a dependent variable is established; and several research hypotheses are proposed on the basis of the two-factor theory, psychological contract theory, and self-efficacy theory. The following conclusions are made through the testing of hypotheses based on questionnaire investigation: Job satisfaction of new generation employees in the new energy industry positively affects the contextual performance; organizational commitment plays a mediating role between job satisfaction and contextual performance; the sense of self-efficacy plays a role of moderating job satisfaction, organizational commitment, and contextual performance. There is a mediated moderation effect and regulated mediation effect in the model. These conclusions are of great significance to the healthy development of new generation employees in the energy industry.

## 1 Introduction

In terms of psychology, the new generation employees in the energy industry are characterized by low labor enthusiasm, poor psychological endurance, diverse value orientation, and strong self-awareness; while in terms of behaviors, they are characterized by high turnover rate, the emphasis on the rapid realization of self-value and the balance between work and life, as well as the resistance to changes. In view of these problems of new generation employees in the energy industry, the literature review generally focuses on research subjects and contextual performance. At present, few scholars have conducted relevant studies on new generation employees in the energy industry, but discussed the development prospects and valuation of enterprises in the energy industry. N. Li and S.X. Li discussed the development trend of the energy industry in the context of strategic transformation. M.Y. Jiang discussed the valuation of the energy industry. As for the discussion of employees in the energy industry, L.L. Zheng studied the relationship between work stress and unsafe behaviors in the energy industry [1], and M.M. Song discussed the performance appraisal forms of employees in the new energy industry. As for the studies related to the new generation employees, domestic scholars have formed several mature research perspectives and carried out detailed research from different moral dimensions. K. Chen, S.Y. Dong and M. Cai respectively studied the turnover behavior or intention of new generation employees from the aspects of performance appraisal fairness and prospect perception. M. Zhu, Q. J. Wang, and H. Chen analyzed and studied the incentives of new generation employees. In conclusion, the research on new generation employees has been a hot topic recently, and research on energy industry employees is rarely involved [2].

For studying job performance, scholars often divide it into multiple dimensions based on different situations. Borman&Motowidlo proposed a two-factor model of job performance on the basis of previous studies, and divided job performance into task performance and contextual performance in combination with the studies on organizational citizenship behaviors and prosocial behaviors. The research of contextual performance involves the dimensions such as attitude, personality, group and civic behaviors, especially the constructs of job satisfaction, organizational commitment, organizational identity, organizational politics perception, work values, organizational fairness concept and sense of self-efficacy. X.X. Xing, J.Z. Liu, and Y.F. Zhang studied the impact of job satisfaction on contextual performance and found that job satisfaction and contextual performance were positively correlated [3]. N. Zhao and J. Du studied the relationship between organizational commitment and contextual performance, and found that they generally showed a positive correlation. As for personality, Z. J. Wang, R. Cao and C.Y. Tang respectively studied the impact of Big Five personality and employee personality on contextual performance. As for the perceptual dimension, Y.L. Jia found that the sense of self-efficacy showed a significant positive impact on contextual performance. Based on previous studies, this paper studies the impact of job satisfaction, organizational commitment and the sense of self-efficacy on contextual performance of new generation employees in the energy industry.

The research on job satisfaction was officially initiated in the early 1930s. Hussey studied the daily emotional rhythms of employees with a mood checklist, and linked these rhythms to daily changes in work efficiency, physiological state, and life events after work. The research on job satisfaction is extensive, which is especially related to enterprise management. Choi studied the relationship between job change, life satisfaction and job satisfaction among the elderly. Henrique studied the relationship between job durability and job satisfaction in artistic career [4]. Steele found that employees' job satisfaction was generally low in the bullying environment [5]. In recent years, job satisfaction has become the main reason for employee behaviors [6]. Subsequent studies mainly focused on the predictors and performance-related factors of satisfaction, the best measurement methods of satisfaction and the development of the theory of satisfaction formation. Vieira studied the relationship between job satisfaction and organizational commitment with survey data [7]. Kwon studied the moderating effect of job satisfaction between job demand and job performance [8]. In conclusion, in the fields related to psychology, job satisfaction is mostly discussed as an outcome of the psychological process; while in the fields of organizational behaviors and business management, scholars mainly take job satisfaction as the antecedent explanation of employee behaviors.

Organizational commitment has become a hot topic in organizational behavior and management. Scholars have studied common constructs in the field of organizational behavior, such as transformative leadership and the sense of organizational support. X.P. Liu, as a leading scholar, analyzed and studied the influencing factors and production reasons of organizational commitment. X. Chen and M. Zhang et al. made a comprehensive review of the antecedents and consequences of organizational commitment. Most scholars believed that the antecedent variables of organizational commitment included job satisfaction, job engagement, motivation, tension, and occupational commitment, etc., and the outcome variables included job performance, alternative job opportunities, job hunting intention, turnover intention, attendance rate, and lateness rate, etc. Subsequent studies on organizational commitment also selected a part of these constructs as the basis or supplement. For example, J. Liu studied the relationship between transformative leadership and organizational commitment, and Son found that self-leadership of employees in a sports center had a positive impact on organizational commitment. Sezen-Gultekin believed that organizational commitment played a mediating role between teachers' emotional labor and job engagement [9]. In conclusion, studies on the antecedents and consequences of organizational commitment have a certain academic value, and there have been relatively extensive studies on the antecedents and consequences of organizational commitment as mediators.

The sense of self-efficacy was derived from the Social Cognitive Theory. Scholars often took it as an antecedent variable or mediator to explain some results in the fields of management and medicine. Reviewing the classic literature articles related to the sense of self-efficacy, S.C. Gao and K. Yao et al. believed that the sense of self-efficacy would be affected by the factors such as the competence-based view, feedback style, cultural factors, emotional characteristics, and organizational heterogeneity, and it would also affect the range and regulatory mechanism of organizational behavior, including variables such as job performance. Z.M. Wang elaborated on the concept of the sense of self-efficacy. Many scholars, including W.B. Zhou, Z.W. Wan, F.F. Wang, Liu, and Liao et al., have studied the relationship between the sense of self-efficacy and variables such as social support, psychological advantage, turnover intention and emotion [10,11]. In conclusion, the sense self-efficacy was taken as both an ante-dependent variable and outcome variable. The discussion of the sense of self-efficacy needs to be further conducted in combination with theoretical analysis.

Although there were studies on contextual performance of new generation employees, they mainly involved the perspective of external characteristics, rather than the psychological state and process of the new generation employees. This paper attempts to explore the mental state process and behavioral characteristics of new generation employees in the energy industry through empirical research, to further explore the impact on contextual performance, and put forward feasible suggestions for employees' mental health from the perspective of enterprise management, thus increasing the employees' contextual performance and work contribution. This paper applies the theory of self-efficacy to organizational behavior and propagates the theory. The integration of self-efficacy theory and social cognition theory has certain novelty and originality in applying to the new generation of employees with fluctuating mental health status. Next, this paper will discuss from three theoretical analysis and hypothesis. Nonetheless, it's crucial to first recognize models that are derived from empirical approaches to organizational and management research that may be helpful. These models aid in our comprehension of the connections between ideas like job happiness, organizational commitment, and self-efficacy. There are many different phenomena that are referred to as mediating or mediating, and it's not simply a straightforward mediating or mediating effect, is that why Chapter 2.1 was written in this study. In addition, it is necessary to go into more detail about the study's methodology, which primarily uses questionnaires to collect data on the younger generation of energy industry employees. The testing of theories is done with Mplus and other analytical tools. This study is associated with numerous quantitative analyses, but it does not include case studies.

## 2 Theoretical analysis and research hypothesis

### 2.1 Research model conception

Before constructing the model and making hypotheses, the required effect is expounded. The independent variable is X, the mediator is M, the dependent variable is Y, and the moderator is W. The mediating effect and moderating effect are analysis models commonly used by scholars. For specific regression equations and testing methods, please refer to Z.L. Wen, and they will not be further introduced [12].

When there is the mediating effect and moderating effect in the model, there may also be other effects. The first case is the moderated mediation effect, in which, the moderator changes the strength of the relationship between the independent variable and the mediator, and then changes the strength of the relationship between the mediator and the dependent variable, thus moderating the entire mediating effect. Z.L. Wen studied the regression equation required for the test, as shown in 3-1 and 3-2.

(3-1) $$M=a_{0}+a_{1}X+a_{2}W+a_{3}XW$$

(3-2) $$Y=c_{0}^{\prime }+c_{1}^{\prime }X+c_{2}^{\prime }W+c_{3}^{\prime }XW+b_{3}M+b_{2}WM$$

Mediated moderating effect (Type 1). Mediated moderation means that the independent variable X affects the dependent variable Y through the mediator M, and the moderating effect operates (at least in part) through the mediator (M). The mediated moderation can be defined as the process that the independent variable X influences the dependent variable Y through the mediator M, and the moderating effect operates (at least in part) through the mediator (M). In this study, this effect is defined as the mediated moderation effect (Type 1), and the regression equations are shown in 3-3 and 3–4. Even if the regression equations are consistent, the mediated moderation and the moderated mediation have different connotations, with different test parameters and steps. The actual steps can be referred to the test method determined by B.J. Ye [13].

(3-3) $$M=a_{0}+a_{1}X+a_{2}W+a_{3}XW$$

(3–4) $$Y=c_{0}^{\prime }+c_{1}^{\prime }X+c_{2}^{\prime }W+c_{3}^{\prime }XW+b_{3}M+b_{2}WM$$

Mediated moderation effect (Type 2). X.P. Chen proposed a second type of mediated moderation effect, namely the mediating of the moderating of the relationship between independent variables and dependent variables by the mediator. The current model should be established in three steps: First, it is necessary to prove that the moderator may have a moderating effect between the independent variable and the dependent variable with specific theories; second, the moderator must have an impact on the mediator; third, the mediator can moderate the relationship between the independent variable and the dependent variable, and transfer the moderating effect of the moderator on the relationship between the independent variable and the dependent variable (Type 2). The required regression equations are shown in 3–5, 3–6, and 3–7. The existence of the mediated moderating effect (type 2) can be tested through determining whether (c3-b5) is significantly non-0.

(3–5) $$Y=c_{0}+c_{1}X+c_{2}W+c_{3}XW$$

(3–6) $$M=a_{1}+a_{2}W$$

(3–7) $$Y=b_{1}+b_{2}X+b_{3}M+b_{4}W+b_{5}XW+b_{6}XM$$

### 2.2 Research hypothesis

#### 2.2.1 Research hypothesis on the relationship between job satisfaction and contextual performance of new generation employees in the energy industry

As stated by Herzberg's two-factor theory, human motivation is the result of the joint action of internal factors, external factors and problem-solving ability; motivators can stimulate individual willingness to work, and then pursue greater work performance. Scholars have studied the antecedent and outcome variables of job satisfaction from multiple perspectives. Employee behaviors can be affected by the factor of satisfaction, and job satisfaction, as such a factor, often induces prosocial behaviors of employees and affects their work efficiency. The arising prosocial behaviors can be extended to the concept of contextual performance in the current organizational behavior theory. In terms of the effect of job satisfaction on work performance, at the individual incentive level, the incentives will improve their job satisfaction and help them make behaviors conducive to enterprise development [14]. In addition to the characteristics of poor psychological endurance and strong self-awareness, the new generation employees in the energy industry will have prosocial behaviors like other employees, which will affect their contextual performance. The environment and treatment that these employees meet make them less satisfied with their jobs, resulting in self-centeredness, less pro-social behaviors and lower contextual performance. In conclusion, employees' job satisfaction has a positive impact on contextual performance in multiple behavior scenarios combined with the two-factor theory. Therefore, the following hypothesis is made.

Hypothesis H1 Job satisfaction of new generation employees in the energy industry positively affects their contextual performance.

#### 2.2.2 Research hypothesis on the relationship between organizational commitment, job satisfaction and contextual performance of new generation employees in the energy industry

Psychological contract refers to the idea of mutual obligations in exchange agreements, involving both the enterprises and employees. Argyris proposed that employment contract and psychological contract formed the basis of the labor-capital relationship. The exchange nature of what is given by both parties, as opposed to what is expected from the other, is crucial, which may be called the "mutuality" of expectations and obligations. Psychological contract can partly explain the reason why employees are willing to stay in an enterprise when they are well paid. Organizational commitment, to some extent, is a one-way psychological contract, which only involves the employees’ emotion towards the organization, and their emotional recognition towards the organization would affect the turnover intention and performance appraisal. Allen explained the reasons why employees are willing to stay in an enterprise from three constitutive dimensions of organizational commitment. Based on the studies of X.P. Liu et al., when employees are well treated in an organization, they would have higher job satisfaction and are willing to make performance behaviors or pro-social behaviors for the organization. Therefore, from the perspective of emotional commitment, organizational commitment can be taken as an outcome variable of job satisfaction; while from the perspective of normative commitment, organizational commitment can be taken as an antecedent variable of contextual performance. For the new generation employees in the energy industry, the poor working environment and atmosphere would result in low job satisfaction and enthusiasm, further affecting their willingness to stay in the organization, making them more self-centered, marginalized in collective activities and work, and unwilling to contribute to the collective.

The specific hypothesis is as follows.

Hypothesis H2 The organizational commitment for new generation employees in the energy industry plays a mediating role between job satisfaction and contextual performance.

#### 2.2.3 Research hypothesis on the relationship between the sense of self-efficacy, job satisfaction, organizational commitment and contextual performance of new generation employees in the energy industry

Bandura proposed the Social Cognition Theory to explain human behaviors and functions from the perspective of "tripartite interaction" of cognition, behavior and environment [15]. The sense of self-efficacy, as a subjective self-judgment of the interaction effect between individuals and the environment, was not proposed without foundation, but on the basis of certain experience or information. The process and results of the interaction between human and environment reveal abundant information of different nature to individuals. The information related to individual interaction efficacy is called self-efficacy information, and self-efficacy is formed through cognitive processing of such information. The processing of such information may be affected by individual attitudes and emotions, which, in turn, will be affected by such information. The sense of self-efficacy of employees can moderate the impact of job satisfaction on organizational commitment. Self-efficacy theory, as an important part of social cognitive theory, mainly emphasizes that individuals' beliefs affect behavior choice, effort level and persistence [16]. Employees' cognition of others' abilities is a key determinant of behavioral outcomes. For employees with different levels of self-efficacy, their attitudes and emotions may produce different effects on behaviors. The behaviors of employees with high self-efficacy would be affected by the sense of self-efficacy. Job satisfaction and organizational commitment may have a lower effect on contextual performance, thus creating a negative moderating effect. Currently, employees in difficult situations may not have the opportunity to acquire mastery experience or the skill of modeling to develop a high level of self-efficacy [17]. As for new generation employees in the energy industry, the low sense of self-efficacy and the lack of confidence make and the impact of job satisfaction, organizational commitment and contextual performance stronger.

Therefore, the following hypotheses are proposed.

Hypothesis H3a The sense of self-efficacy of new generation employees in the energy industry has a negative moderating effect on the relationship between job satisfaction and organizational commitment.

Hypothesis H3b The sense of self-efficacy of new generation employees in the energy industry has a negative moderating effect on the influence between organizational commitment and contextual performance.

Hypothesis H3c The sense of self-efficacy of new generation employees in the energy industry has a negative moderating effect on the influence between job satisfaction and contextual performance.

Self-efficacy theory emphasizes the importance of individual factors, and acknowledges the profound impact of behavioral and environmental factors on outcomes. The Triadic Reciprocal Determinism further reinforces the idea that if the effects of the environment are consistent (i.e., a level playing field for all), the self-efficacy belief will play a greater role in determining human behaviors and ultimately shaping outcomes. As inferred above, self-efficacy could moderate job satisfaction, organizational commitment and contextual performance. Due to the mediating effect of organizational commitment, it can be inferred that the intervention of the moderator of self-efficacy affects the original mediating effect of organizational commitment, and then moderates the relationship between the mediator and outcome variable. The new generation employees in the energy industry with a strong sense of self-efficacy have increasingly strong contextual performance, and the influence path of job satisfaction is no longer mediated by organizational commitment, but directly affects contextual performance.

Therefore, the following hypothesis is proposed.

Hypothesis H3d There is a moderated mediation effect.

Moderated mediation and mediated moderation exist simultaneously in many scenarios. According to the self-efficacy theory, the experience of successful performance can lead to stronger effects. The perception of self-efficacy through successful performance depends on various individual and situational factors. In the constructed model, there is significant influence of job satisfaction and self-efficacy of the new generation employees in the energy industry, and also a strong correlation between job satisfaction and organizational commitment, as well as self-efficacy and organizational commitment [18]. There are interactive variables of job satisfaction and self-efficacy that influence the mediating effect. The sense of self-efficacy has a moderating effect between job satisfaction and contextual performance, and between job satisfaction and organizational commitment. Therefore, the moderating of the sense of self-efficacy is dispersed by mediators, forming indirect moderating and direct moderating.

Therefore, the following hypothesis is proposed.

Hypothesis H3e There is a mediated moderation effect (Type 1).

According to the self-efficacy theory, the two key factors determining behaviors are the sense of self-efficacy and outcome expectation. Individual evaluation is closely associated with affective dimension, and the sense of self-efficacy is associated with organizational commitment, thus affecting the entire mediation model, and forming a complete moderating model. However, the review of literature articles and theories found that few scholars explained and supported the impact of the interaction between the sense of self-efficacy and organizational commitment on the entire effect. Therefore, there is no mediated moderation effect in the model (Type 2).

Therefore, the following hypothesis is proposed.

Hypothesis H3f There is no mediated moderation effect (Type 2).

#### 2.2.4 Summary of research models and hypotheses

**Fig. 1:**
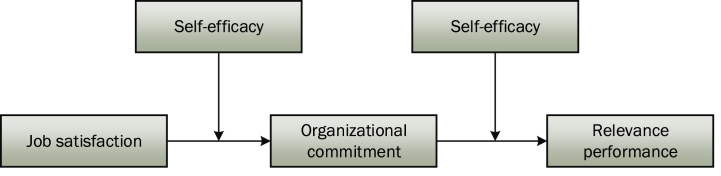
Theoretical research model.

In combination with literature articles and theories, this section explains the relationship construction among independent variables, dependent variables, mediators and moderators. The relevant hypotheses of job satisfaction, organizational commitment, contextual performance and the sense of self-efficacy are summarized (Table 1), and the model is shown in Fig. 1.

**Table 1:** Summary of research hypotheses.

Original hypothesis		Contents
H1		Job satisfaction of new generation employees in the energy industry positively affects contextual performance
H2		The organizational commitment for new generation employees in the energy industry plays a mediating role between job satisfaction and contextual performance
H3		
	H3a	The sense of self-efficacy of new generation employees in the energy industry has a negative moderating effect on the relationship between job satisfaction and organizational commitment
	H3b	The sense of self-efficacy of new generation employees in the energy industry has a negative moderating effect on the influence between organizational commitment and contextual performance
	H3c	The sense of self-efficacy of new generation employees in the energy industry has a negative moderating effect on the relationship between job satisfaction and contextual performance
	H3d	There is a moderated mediation effect
	H3e	There is a mediated moderation effect (Type 1)
	H3f	There is no mediated moderation effect (Type 2)

## 3 Variable data source and measurement

According to the research hypothesis and the research purpose, a suitable scale was selected for design, and small sample data were pre-processed to adjust the questionnaire. In the research framework, conventional variables such as gender, education, age and years of work were designed as the top part of the questionnaire, and the main body of the questionnaire was composed of job satisfaction scale, organizational commitment scale, contextual performance scale and self-efficacy scale.

### 3.1 Questionnaire design

Job satisfaction is influenced by many factors. Most scholars use the Minnesota Scale in their studies. This study uses this scale item to modify part of the scale and delete "social service" and other options according to the characteristics of the new generation employees in the energy industry such as poor psychological endurance. The revised scale is divided into two dimensions of internal satisfaction and external satisfaction. The use of some items is more suitable for the working environment and remote working area of the new generation employees in the energy industry, with a total of 20 questions. The topic distribution is shown in Table 2.

**Table 2:** Job satisfaction questionnaire.

Construct dimension	Number of questions	Meaning description
Intrinsic satisfaction	11	Ability, accomplishment, independence, security, responsibility, etc
External satisfaction	9	Leadership, relationship with colleagues, working conditions, cultural atmosphere, etc

Many mature scales have been developed for the study of organizational commitment in academic circles. Domestic scholar Liu Xiaoping divided organizational commitment into emotional commitment, continuous commitment and normative commitment, and established an organizational commitment questionnaire in line with the domestic situation. Based on the high-quality scale of Allen, Liu Xiaoping, Ma Ling and other scholars, this paper investigates the problem and applies it to the new generation employees in the energy industry. The topic distribution is shown in Table 3.

**Table 3:** Organizational commitment questionnaire.

Construct dimension	Number of questions	Meaning description
Emotional commitment	8	Employee's emotional dependence, identification and investment in the organization
Ongoing commitment	5	The employee's perception of the loss caused by leaving the organization
Normative commitment	5	Employee's sense of commitment to remain with the organization

Van Scotter divided contextual performance into two dimensions, namely, job dedication and interpersonal promotion. Van's questionnaire was in line with the personality characteristics and behavioral characteristics of the new generation employees in the energy industry. Combined with the two-dimension contextual performance scale given by Wu Xiaoliang and Yan Yanyan, a scale of 14 questions was set in this study. The topic distribution is shown in Table 4.

**Table 4:** Contextual performance questionnaire.

Construct dimension	Number of questions	Meaning description
Interpersonal facilitation	7	Interpersonal tendencies that contribute to the achievement of goals
Job dedication	7	Take initiative to solve problems at work

German psychologist Ralf and his colleagues began to compile the general self-efficacy scale in 1998. Domestic scholars Wang Caikang, Hu Zhongzhong and Liu Yong studied the reliability and validity of the Chinese version of the scale. The revised questionnaire had high reliability and good predictive validity, and confirmed its feasibility and reliability. In this study, the scale translated into Chinese by Wang Caikang was adopted for measurement, and some items were modified to meet the psychological characteristics of the new generation employees in the energy industry. The topic distribution is shown in Table 5.

**Table 5:** Self-efficacy questionnaire.

Construct dimension	Number of questions	Meaning description
Self-efficacy level	3	The level at which an individual feels able to perform the behavior required for the activity task
Self-efficacyintensity	4	The individual's level of confidence in completing activities or tasks of varying degrees of difficulty and complexity
Self-efficacy beadth	3	The strength of self-efficacy can be extended to other similar behaviors or situations

### 3.2 Release and collection of questionnaires

After the design of the questionnaire is completed, the distribution of the questionnaire is planned. The first is the total number of samples, the questionnaire selects 95 % confidence level(Z), accepts 5 % error range(E), and the proportion of occurrence of a certain feature in the population is 0.5(p). According to the sample size calculation formula (4-1), the optimal value of the sample size can be obtained as 385, which is about 322 after modification by the overall correction factor. Assuming that the expected response rate of the questionnaire is 80 %, the optimal value of the sample size is about 402.5. Therefore, 400 is selected as the best sample size. A quarter of the formal sample size is selected as a pre-survey to test the reliability and validity of the questionnaire. In terms of sampling technology, simple random sampling is adopted, because the sampled groups are the new generation employees, the use of simple random sampling is fairer and more efficient. In terms of data collection process, the "questionnaire star platform" is used to find the new generation of energy employees, and then individuals are selected by simple random sampling to publish questionnaires and recover data.

(4-1) $$n=\frac{Z^{2}\times p\times \left(1-p\right)}{E^{2}}$$

This paper studied and analyzed the psychological state and behavioral characteristics of the new generation employees in the energy industry. Informed consent of the participants was obtained for this study. A total of 100 questionnaires were sent to the new generation employees in the energy industry through field investigation and online questionnaire distribution. Some questionnaires that did not meet the characteristics of the new generation employees in the energy industry were excluded, and 74 valid questionnaires remained. After the pre-test, the research found that the questionnaire met the requirements of the expected test. Some items that did not meet the requirements were modified, and the scale had no significant changes overall. Therefore, the formal questionnaire consisted of five parts: basic information, job satisfaction, organizational commitment, contextual performance and self-efficacy. 400 formal questionnaires were issued to the new generation of post-90s employees in the energy industry, and 309 valid questionnaires were recovered.

### 3.3 Validity and reliability testing of questionnaires

The reliability test of each scale is shown in Table 6. Cronbach's Alpha(α) coefficient results show that Cronbach's Alpha(α) coefficient of job satisfaction scale, organizational commitment scale, contextual performance scale and self-efficacy scale are all above 0.9, and the lowest self-efficacy coefficient is 0.937. Therefore, the reliability quality of the research data is high, and the reliability test of the questionnaire passes.

**Table 6:** Reliability test results of the questionnaires.

Questionnaire name	Cronbach's α coefficient
Job satisfaction	0.965
Organizational commitment	0.962
Contextual performance	0.947
Self-efficacy	0.937

All the scales used in this study are mature scales, which have been used in many industries. In the process of collecting small sample data, we did not receive feedback of unclear meaning and repeated items, so we did not test the content validity. Before validity test, exploratory factor analysis was performed on the scale using SPSS 26 to check whether the scale was suitable for the next test.

KMO values and cumulative variance interpretation rates of the four questionnaires are shown in Table 7. According to the analysis in the table, KMO values of job satisfaction scale, organizational commitment scale, contextual performance scale and self-efficacy scale are all above 0.9, and the cumulative variance explanation rate is above 60 %, which means that the information of research items can be effectively extracted.

**Table 7:** KMO values and cumulative variance interpretation rate of the four questionnaires.

Questionnaire name	KMO value	Explanation rate of cumulative variance	Sig
Job satisfaction	0.980	69.988 %	0.000
Organizational commitment	0.982	75.076 %	0.000
Contextual performance	0.974	71.147 %	0.000
Self-efficacy	0.959	77.913 %	0.000

After KMO value and cumulative variance explanation rate test, confirmatory factor analysis was performed on the questionnaire using Mplus. Table 8 shows the test results of the four scales. According to Wang Mengcheng (2014), confirmatory factor analysis verification criteria for mplus was proposed (strict standard: χ2/df ≤ 3; RMSEA≤0.05; CFI≥0.95; TLI≥0.95; SRMR≤0.05). The confirmatory factor analysis of the four questionnaires passed, and the validity of the questionnaires was tested.

**Table 8:** Results of confirmatory factor analysis of the four questionnaires.

Questionnaire name	RMSEA	CFI	TLI	Chi-Squared Test	degree of freedom	SRMR
Job satisfaction	0.044	0.963	0.959	271.552	169	0.047
Organizational commitment	0.000	1.000	1.002	125.751	132	0.021
Contextual performance	0.000	1.000	1.007	61.018	76	0.018
Self-efficacy	0.017	0.998	0.998	34.857	32	0.018

## 4 Empirical analysis's result

### 4.1 Correlation analysis

A correlation analysis was conducted between demographic variables and job satisfaction, organizational commitment, contextual performance and self-efficacy, and the results were shown in Table 9. It can be seen from the table that gender, age, educational background and working years have no significant correlation with job satisfaction, organizational commitment, contextual performance and self-efficacy at the level of 0.01. This indicates that gender, age, education background and working years of the new generation employees in the energy industry are not influential factors for job satisfaction, organizational commitment, contextual performance and self-efficacy, and are not used as control variables in the subsequent test.

**Table 9:** Correlation analysis results of four variables including demographic variables and job satisfaction.

Questionnaire name	Gender	age	education	years of work
Job satisfaction	0.034	−0.022	−0.026	0.013
Organizational commitment	0.048	−0.037	.-0.019	0.016
Contextual performance	0.064	−0.066	0.035	−0.011
Self-efficacy	0.021	−0.049	−0.001	−0.007

**Fig. 2:**
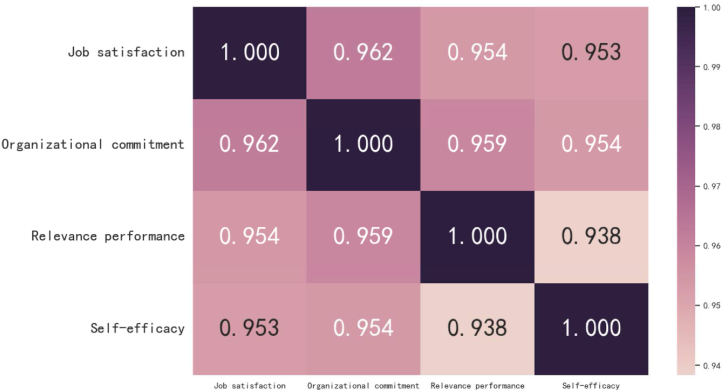
Correlation analysis results of job satisfaction, organizational commitment, contextual performance, and self-efficacy.

The correlation analysis results of job satisfaction, organizational commitment, self-efficacy and contextual performance are shown in Fig. 2. The four variables of job satisfaction, organizational commitment, self-efficacy and contextual performance show significant positive correlation at the level of 0.01, and the correlation between the variables is very strong. According to the theory, there is indeed a strong correlation between job satisfaction, organizational commitment and contextual performance. Contextual performance has a strong correlation with job satisfaction and organizational commitment, while the correlation with self-efficacy is slightly weak.

### 4.2 Hypothesis testing

The regression equations required for hypothesis testing were sorted out, and there were 8 models. Model 1 tested the main effect, models 2 and 3 tested the mediating effect of organizational commitment, models 4–6 tested the moderating effect of self-efficacy, models 4 and 7 tested the moderating effect of the moderated and mediated (type 1), and models 6 and 8 tested the moderating effect of the mediated (type 2). Run Mplus software to test the fitting results of each model and the required parameter test results, as shown in Table 10 and Table 11. In order to verify the expression, the four variables of job satisfaction, organizational commitment, contextual performance and self-efficacy were simplified as js, oc, rp and se.

**Table 10:** Fitting results of each model.

Model	R	R^2^	Adjusted R^2^	S.E.	F	Sig
Model 1	0.954^a^	0.911	0.910	0.27395	3130.390	0.000^b^
Model 2	0.962^a^	0.926	0.926	0.25176	3843.283	0.000^b^
Model 3	0.966^a^	0.933	0.933	0.23774	2129.129	0.000^b^
Model 4	0.971^a^	0.943	0.943	0.22084	1696.314	0.000^b^
Model 5	0.964^a^	0.930	0.929	0.24308	1346.384	0.000^b^
Model 6	0.961^a^	0.924	0.923	0.25384	1232.314	0.000^b^
Model 7	0.968^a^	0.936	0.935	0.23278	891.562	0.000^b^
Model 8	0.969^a^	0.940	0.939	0.22278	944.562	0.000^b^

**Table 11:** Test results of each model parameter.

Model	Multiple Regression	Detection parameter	Result	Sig
Model 1	rp = a+b*js	b	0.948	0.000
Model 2	oc = a+b*js	b	0.962	0.000
Model 3	rp = a+b1*js + b2*oc	b1，b2	0.424，0.543	0.000，0.000
Model 4	oc = a+b1*js + b2* se + b3*(js * se)	b3	−0.143	0.000
Model 5	rp = a+b1* oc + b2* se + b3*(oc* se)	b3	−0.173	0.000
Model 6	rp = a+b1* js + b2* se + b3*(js * se)	b3	−0.177	0.000
Model 7	rp = a+b1*js + b2* se + b3*(js * se) + b4* oc + b5*(oc * se)	b4，b5	0.829，-0.114	0.000，0.048
Model 8	rp = a+b1* js + b2* oc + b3* se + b4*(js * se) + b5*(oc *se)	b4	0.023	0.639

In the fitting results table, model 1 represents the regression equation of job satisfaction and contextual performance, and the significance is infinitely close to 0, indicating that there is a linear regression relationship between job satisfaction and contextual performance, and the adjusted R2 is 0.910, indicating that job satisfaction can explain 91 % of the variation of contextual performance. In the parameter test results, the VIF value of the test parameter is less than 3, and its coefficient is 0.948, so the regression equation is as follows:

Contextual performance = 0.165 + 0.948* Job satisfaction.

Therefore, it can be proved that job satisfaction of the new generation of H1 energy industry employees has a positive impact on contextual performance.

Further verify the regression of job satisfaction to organizational commitment, and the multiple regression of contextual performance to organizational commitment and job satisfaction. In the fitting result model 2, the significance of the regression equation is infinitely close to 0, indicating that there is a linear regression relationship between job satisfaction and contextual performance, and the adjusted R2 is 0.926, indicating that job satisfaction can explain 92.6 % of the variation in contextual performance. In the parameter test results, the VIF value of the test parameter is less than 3, and its coefficient is 0.966, so the regression equation is as follows:

Organizational commitment = 0.110 + 0.966* Job satisfaction.

In model 3 of the fitting results, the significance of the regression equation is infinitely close to 0, indicating that there is a linear regression relationship between job satisfaction and contextual performance, and the adjusted R2 is 0.933, indicating that job satisfaction can explain 93.3 % of the variation in contextual performance. In the parameter test results, the coefficient of job satisfaction is 0.424 and the coefficient of organizational commitment is 0.543. Therefore, the regression equation is as follows:

Contextual performance = 0.106 + 0.424* Job satisfaction +0.543* Organizational commitment.

The intermediary effect value can be obtained by multiplicating the coefficient of organizational commitment in model 2 (0.543) and the regression coefficient between job satisfaction and organizational commitment in model 3 (0.966). By adding the coefficient of job satisfaction in model 3 (0.424), the result is 0.948, equal to the total effect coefficient in model 1. It can be seen that hypothesis H2 is valid. The intermediate effect accounted for 55.33 % of the total effect.

In the fitting result model 4, the P value of interaction item * self-efficacy is 0.000, and the R2 of interaction item has a significant change compared with that of interaction item, indicating that self-efficacy has a significant moderating effect on the relationship between job satisfaction and organizational commitment. Assuming the H3a test is valid, the regression equation with interaction item added is as follows:

Organizational commitment = -0.056 + 0.996* Job satisfaction +0.832* self-efficacy −0.143* (job satisfaction * self-efficacy)

In the fitting result model 5, the P value of the interaction item organizational commitment * self-efficacy is 0.000, and the R2 of the interaction item has a significant change compared with that of the interaction item, indicating that self-efficacy plays a significant moderating role in the relationship between organizational commitment and contextual performance. Assuming the H3b test is valid, the regression equation with the interaction item added is as follows:

Contextual performance = -1.243 + 1.210* Organizational commitment +0.786* self-efficacy −0.173* (organizational commitment * self-efficacy).

In the fitting result model 6, the P value of interaction item * self-efficacy is 0.000, and the R2 of interaction item has a significant change compared with that of interaction item, indicating that self-efficacy plays a significant moderating role in the relationship between job satisfaction and organizational commitment. Assuming the H3c test is valid, the regression equation with interaction item added is as follows:

Contextual performance = -1.275 + 1.163* Job satisfaction +0.852* self-efficacy −0.177* (job satisfaction * self-efficacy)

Examine the mediated moderation effect (Type 1). In the fitting result model 7, the coefficient of organizational commitment (0.829) and job satisfaction * self-efficacy (−0.143) in model 4 both show a significance of 0.000, indicating that there is a moderated mediating effect, assuming that the H3d test is valid. The coefficient of organizational commitment * self-efficacy in model 7 (−0.114) and job satisfaction in model 4 (0.996) was significant at 0.05 level, and job satisfaction * self-efficacy (−0.027) was not significant, indicating that there was mediated mediation (type 1), and there was completely mediated mediation. Suppose the H3e test holds. The regression equation with interaction terms is as follows:

Contextual performance = -1.027 + 0.419* Job satisfaction +0.565* self-efficacy −0.027* (job satisfaction * self-efficacy) +0.829* Organizational commitment −0.114* (organizational commitment * self-efficacy)

Examine the mediated moderation effect (Type 2). The moderating effect of self-efficacy on job satisfaction and contextual performance and the moderating effect of self-efficacy on job satisfaction and organizational commitment were tested in the previous paper. In the fitting result model 8, The coefficient of self-efficacy * job satisfaction (0.023) and the coefficient of self-efficacy * job satisfaction in model 6 (−0.177) are not reduced to 0 but not significant, indicating that there is no mediated effect (type 2), assuming that the H3f test holds. The regression equation with interaction terms is as follows:

Contextual performance = -2.052 + 1.175* Job satisfaction +1.392* Organizational commitment −0.004* self-efficacy −0.023* (job satisfaction * self-efficacy) −0.290* (organizational commitment * Job satisfaction)

### 4.3 Summary of hypothesis testing results

In this study, samples were collected by questionnaire and data were processed by SPSS26.0 and Mplus8 software. The results showed that the questionnaire could truly reflect latent variables and had good reliability and validity. Hypothesis testing results were obtained through further analysis and processing of the data. The test results are summarized in Table 12.

**Table 12:** Summary of research hypothesis test results.

Hypothesis		Content description	result	Instructions
H1		Job satisfaction of new generation employees in the energy industry has positive influence on contextual performance	establish	
H2		The organizational commitment of the new generation employees in the energy industry plays an intermediary role between job satisfaction and contextual performance	establish	
H3				
	H3a	The self-efficacy of new generation employees in the energy industry has a negative moderating effect on the relationship between job satisfaction and organizational commitment	establish	
	H3b	The self-efficacy of new generation employees in the energy industry has a negative moderating effect on the influence between organizational commitment and contextual performance	establish	
	H3c	The self-efficacy of new generation employees in the energy industry has a negative moderating effect on the relationship between job satisfaction and contextual performance	establish	
	H3d	Moderated mediation effect	establish	
	H3e	Mediated moderation effect (Type 1)	establish	Perfect mediation
	H3f	No mediated moderation effect (Type 2)	establish	

## 5 Discussions and conclusions

### 5.1 Discussions

Firstly, job satisfaction of new generation employees in the energy industry has a positive impact on contextual performance. High job satisfaction is generally associated with higher job performance, which can be affected by multiple factors, such as the work environment, job tasks, and employees' personal characteristics. From the perspective of two-factor theory, new generation employees in the energy industry are willing to help other colleagues, which is conducive to the unity and development of enterprises. The association between job satisfaction and contextual performance is reflected in the fact that job satisfaction can promote employees’ motivation and engagement, thereby improving their performance.

Secondly, the organizational commitment for new generation employees in the energy industry has a mediating effect between job satisfaction and contextual performance. The higher job satisfaction of new generation employees in the energy industry can lead to higher loyalty and commitment to the organization, which may affect the contextual performance. In terms of the structural components of the variables, organizational commitment and contextual performance are similar concepts, with consistency at some levels. However, the overall analysis shows that organizational commitment belongs to the emotional dimension, while contextual performance belongs to the behavioral dimension. Organizational commitment can promote the improvement of employee performance, and the employees can feel the organization's care and support, as a result, they will work harder. In addition, organizational commitment can promote employee innovation and teamwork, thus improving the performance of the entire team. The extent of this correlation varies by industry, culture, and individual differences.

Thirdly, the sense of self-efficacy has a negative moderating effect on job satisfaction, organizational commitment and contextual performance. Social cognitive theory emphasizes that cognition, environment and behavior are interactive. The employees with a high sense of self-efficacy can cope with challenges and difficulties more easily; in addition, they are more confident to succeed in the organization, and align with the values and culture of the organization more easily. For the new generation of energy industry employees with a higher sense of self-efficacy, the individual attitude will be less likely to influence emotions, which will further weaken the influence of emotions on behaviors. On the contrary, there is no close relationship between job satisfaction, organizational commitment and contextual performance, which may result in a negative moderating effect.

Fourthly, there is a moderated mediation effect in the model, and the mediation effect of organizational commitment is influenced by the sense of self-efficacy. There is a totally mediated moderation effect, and the moderation of the sense of self-efficacybetween job satisfaction and contextual performance is achieved by the mediators. There is no direct moderation between self-efficacy and job satisfaction and contextual performance. There is no influence of the sense of self-efficacy on organizational commitment in the model, but a moderating effect between job satisfaction and contextual performance.

Compared with Zhang (2023), Liu (2018) and Hou (2014) el al., this paper first discusses the innovation of the research subjects, i.e. new generation employees in the energy industry, which have not been studied separately by previous scholars. Secondly, it discusses the theoretical innovation. As studied by Wei (2004) and Sun (2006) el al., the integration of two-factor theory and psychological contract theory is reflected in a lot. In this study, self-efficacy theory is added to the model, which is novel. Thirdly, as for the novelty of model construction, Tourism (2013), Oberoi (2015) and Bishakha (2017) et al. only realized the test of mediation effect or moderation effect, while this study discusses both of the two effects menthoed above, as well as the possible mediated moderation and moderated mediation. The model construction is innovative.

Energy enterprises can integrate the management of employees' personality traits, generate temperament reports through self-efficacy surveys and regular interviews at the time of entry, implement enterprise management activities based on different temperaments, and provide tailored activities to enhance internal connections between individuals and the organization. Collaborating with universities and research institutes to conduct training and career guidance for diverse employees, guiding them in making reasonable career plans, leveraging their initiative to reconcile personal and organizational values, enhancing their sense of self-efficacy, and promoting the new generation of employees to develop careers that align with both personal values and organizational goals.

### 5.2 Conclusions

On the basis of the social cognitive theory, this paper analyzes the job satisfaction, organizational commitment, the sense of self-efficacy and management performance of new generation employees in the energy industry, and believes that the employees' contextual performance will be affected by various factors such as the sense of self-efficacy and job satisfaction. Cognition will affect decision-making, and decision-making will have feedback effects on the factors such as cognition and emotion, and reveal the mechanism of inner psychological process of new generation employees in energy industry. As for the social cognitive theory, this paper takes new generation employees as the research subjects. As for the research method, the traditional social cognitive research mostly extracts data through questionnaires. Without being limited to the questionnaire-based method, it combines the experimental method to build a hypothesis model and obtain variable data, so as to multiply and deepen the social cognitive theory. In terms of research methods, this study obtained variable data through questionnaire-based survey to verify the hypothesis, so as to enrich and deepen the social cognitive theory. This study explores the influencing factors of management performance of new generation employees. It is proposed that the moral training of new generation employees should be conducted based on the external behavior quality of employees, and it can also strengthen the mental health education of employees, build a good working environment and trust atmosphere, improve the self-efficacy of new generation employees, avoid uncomfortable emotions, provide direction for the research on the mental health of new generation employees, and provide references for the cultivation of moral quality of enterprises. There are also some limitations in this study: (1) The questionnaire-based survey only comes from some enterprises, the diversity of the sample is insufficient, and the data are insufficient, which limits the credibility of the samples to a certain extent. Follow-up studies can expand the size of the sample to investigate a larger area. Follow-up studies can expand the size of the sample and carry out the experiment in a larger scope. (2) The framework of questionnaire-based survey adopted in this paper is only the questionnaire survey method, and the research method needs to be improved. (3) This survey does not have sufficient changes in environmental factors such as research variables, and the contents of the research need to be expanded.

## Ethics statement

This study was reviewed and approved by Institutional Review Board of School of Economics and Management Southwest Petroleum University, with the approval number: 2023004.

All participants/patients (or their proxies/legal guardians) provided informed consent to participate in the study.

All participants/patients (or their proxies/legal guardians) provided informed consent for the publication of their anonymised case details and images.

## Data availability statement

The data that has been used is confidential.

## CRediT authorship contribution statement

**Hu Xiao:** Methodology, Conceptualization. **Wei Xie:** Writing – original draft, Data curation. **Bo Liu:** Writing – review & editing, Software.

## Declaration of competing interest

The authors declare that they have no known competing financial interests or personal relationships that could have appeared to influence the work reported in this paper.
